# PVDF Nanofiber Membranes for Dissolved Methane Recovery from Water Prepared by Combining Electrospinning and Hot-Pressing Methods

**DOI:** 10.1021/acspolymersau.5c00021

**Published:** 2025-06-20

**Authors:** Félix Montero-Rocca, Jose D. Badia-Valiente, Ramón Jiménez-Robles, Vicente Martínez-Soria, Marta Izquierdo

**Affiliations:** Research Group in Materials Technology and Sustainability (MATS), Department of Chemical Engineering, School of Engineering, 16781 University of Valencia, Avda. Universitat s/n, 46100 Burjassot, Spain

**Keywords:** electrospinning, membrane, electrospun nanofiber membranes, hot pressing, decarbonization, methane recovery

## Abstract

Polyvinylidene fluoride (PVDF) electrospun nanofiber membranes (ENMs) could potentially be used in membrane contactors (MCs) for environmental applications, such as the removal of dissolved CH_4_ from anaerobic effluents. In this work, a PVDF flat-sheet ENM fabrication protocol, including the electrospinning processing and the subsequent hot-pressing treatment (HP), has been developed to produce hydrophobic membranes with suitable integrity and pore size distribution for gas–liquid separations in MCs. The HP study explored the effects of pressure (1, 10, and 20 MPa), temperature (25, 60, 80, and 120 °C), and time (2, 4, 6, and 10 min) on the morphological properties and hydrophobicity of the membranes. Our research revealed that fibers in the PVDF ENMs began to sinter at temperatures above 60 °C when hot-pressed between 1 and 20 MPa. ENM samples were prepared at different dope compositions (10–15% PVDF, 0.00–0.043% LiCl). After HP (≥1 MPa, ≥60 °C, and 6 min), the membrane thickness and water contact angle (WCA) decreased considerably, and lower pore sizes with narrower distributions were obtained. At higher pressure (10 MPa), a noticeable decrease in thickness (from 270 to 38 μm) and WCA (from 139 to 110°) was observed. Additionally, pore size distribution shifted toward a predominant narrow peak of around 0.40 μm. HP enhanced the uniformity of the PVDF crystalline structure without altering its overall crystallinity degree (40–42%). The HP ENM exhibited a comparable dissolved CH_4_ recovery performance to a commercial PVDF membrane and demonstrated sufficient mechanical integrity to endure operating conditions, maintaining a stable performance for at least 80 h.

## Graphical abstract

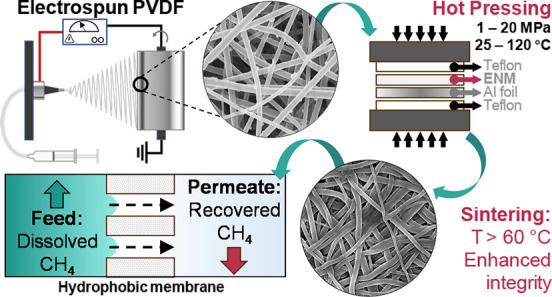

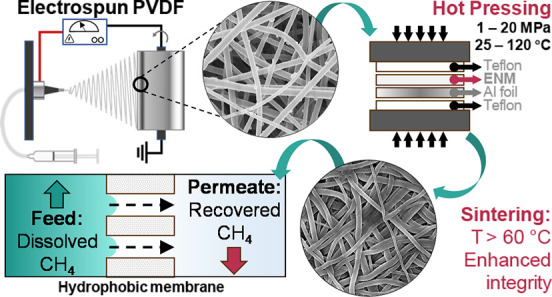

## 1 Introduction

Anaerobic digestion technology is present in several industrial processes,^1,2^ particularly in areas focused on waste management,^3^ wastewater treatment,^4^ renewable energy production,^5,6^ and sustainable agriculture.^7,8^ It converts organic matter into biogas and valuable byproducts, contributing to carbon emission reductions. However, fugitive CH_4_ emissions from anaerobic reactor effluents, which contain substantial concentrations of dissolved CH_4_ due to its partial dissolution in the digestate, are one of the environmental concerns associated with such a technology.^9–13^

Membrane contactors have been successfully employed in the recovery of greenhouse gases through gas absorption, such as in CO_2_ capture from flue gas or biogas,^14,15^ or CH_4_ stripping from aqueous solutions.^13,16^ Thus, membrane contactors were demonstrated to be technically and economically viable technology to capture the dissolved CH_4_ from anaerobic digestor effluents.^9,10,12,17–20^ The process involves gas desorption from the liquid feed through membrane pores, assisted by a sweeping gas stream or vacuum to sustain the mass transfer driving force, while a hydrophobic membrane separates the liquid and gas phases.

Poly­(vinylidene fluoride) (PVDF) stands out among the most common membrane materials for gas–liquid separations in contactors (polysulfone, poly­(ether sulfone), polypropylene, and polydimethylsiloxane)^21–23^ due to its superior chemical resistance, thermal stability, and favorable mechanical properties.^21^ PVDF excels in applications requiring long-term stability and wetting resistance, due to its inherent hydrophobicity, thermal and mechanical robustness, and chemical resistance to acids, bases, organic solvents, oils, and fats.^23^ Commercial PVDF membranes fabricated by conventional methods have been successfully applied in dissolved methane recovery from liquid effluents in membrane contactors, both in their pristine form and with enhanced hydrophobicity through organofluorosilanation.^13,24,25^

Traditional fabrication techniques, such as vapor-induced phase separation, nonsolvent-induced phase separation, track-etching, and sintering often rely on complex control of polymer–solvent interactions, thermal transitions, or chemical reactions to achieve desired porosity, thickness, crystallinity, and morphology.^26–28^ These approaches typically require careful kinetic regulation of phase separation^29,30^ and often involve extensive solvent use or nonmechanical physical processing steps that can limit scalability and structural precision.^31^ In contrast, electrospinning (ESP) offers a more accessible and versatile route to membrane fabrication. It is renowned for creating highly porous nanofibrous structures with precise fiber morphology control and a high ratio of surface area to volume.^28,32^ In ESP, an external electric field induces the formation and elongation of a viscoelastic jet derived from a solution.^32^ The electric charges shape dope into Taylor’s cone, extending the polymer jet into a straight line; electrical bending instability grows as the jet thins and finally solidifies and accumulates as fibers on a grounded collector.^33^ Electrospun nanofiber membranes (ENMs) are a key research focus in sensors, electronics, catalysis, drug delivery, tissue engineering,^34–37^ fuel cells,^38^ and packaging,^39^ among other fields. However, their development as membranes for environmental technologies remains limited, primarily focusing on filtration,^34,35,40^ with less emphasis on gas recovery.^9–11,18,24^ This presents an innovation niche for this production technique.

A wide range of variables affects the production of ENMs, including ambient parameters (room temperature and humidity), process parameters (voltage, tip-to-collector distance, and feed rate), and solution parameters (viscosity, surface tension, and conductivity).^41–44^ On the other hand, additives have been proven to successfully confer desirable properties on either the ESP dope solution or the resulting membrane. For instance, lithium nitrate (LiNO_3_) and lithium chloride (LiCl) are frequently added to PVDF ESP solutions to promote nanofiber formation by increasing solution conductivity and boosting the charge density on the polymer jet surface,^34,45–47^ affecting the nanofiber diameter.^45^ Even under optimal fabrication conditions, nonwoven ENMs often exhibit limited fiber cohesion, resulting in poor mechanical integrity during operation, leading to deformation, tearing, or reduced durability under prolonged stress or high pressure.^41,48,49^

Postfabrication strategies are often implemented to improve the structural properties of ENMs and prevent possible mechanical failure.^41^ The most renowned approaches include cross-linking,^50–53^ drawing or stretching,^54–56^ solvent welding,^41,48,57–60^ heat treatments (HT) at low pressures referred to as annealing,^61–65^ and HT at higher pressures referred to as hot-pressing (HP).^41^ HT is typically conducted at temperatures ranging from the polymer crystallization temperature to its melting point,^41,63,66^ improving the crystallinity and promoting interfiber welding through partial fiber fusion in ENMs.^41^ This is achieved through HP by subjecting a polymer to specific pressure and temperature conditions.^41^ The enhanced structural integrity of ENMs improves the overall mechanical strength and increases the fiber diameter while reducing its thickness, porosity, and bubble point,^41,67^ without introducing additional solvents or hazardous substances, greening the ENMs manufacturing process. For ENMs, HT involves the use of glass plates,^45,68,69^ where the sample experiences pressures below 2 kPa,^70^ and HP-specialized setups,^71^ especially when higher pressures are required, reaching up to 20 MPa or even higher.^67,72^ PVDF ENMs are usually processed at temperatures ranging from 25 to 170 °C, which encompasses the glass transition range of this thermoplastic material and prevents approaching its melting point of approximately 177 °C.

This study aims to determine the fabrication conditions for preparing hydrophobic flat-sheet PVDF ENMs especially designed for the recovery of dissolved CH_4_ from anaerobic effluents with membrane contactors. Different ESP dopes and HP post-treatment conditions were explored to enhance the mechanical and surface properties of the membranes. The effects of HP pressure, temperature, and time on the hydrophobicity and the morphological properties of the PVDF ENMs, were evaluated. Finally, the operational performance of the HP PVDF ENMs was compared with that of a commercial membrane in the recovery of dissolved CH_4_ from water in a flat-sheet membrane contactor under sweep gas operation. To the best of our knowledge, electrospun PVDF membranes have not been previously reported for dissolved CH_4_ recovery from anaerobic effluents or aqueous streams, thus motivating the current investigation into their potential for this application.

## 2 Materials and Methods

### 2.1 PVDF Membrane Fabrication by Electrospinning

To prepare the ESP dopes, PVDF powder (Kynar 761, Arkema, France) was dissolved in a 6:4 v/v dimethylformamide (DMF, VWR, 98% purity, USA) and acetone (VWR, ACS grade, USA) mixture in a Pyrex bottle under 60 °C and 175 rpm orbital agitation overnight. Three polymer concentrations were studied: 10, 12, and 15 wt %. The addition of LiCl (ThermoScientific, anhydrous, 98+%, USA) at a 0.43 mg_LiCl_ g_PVDF_^–1^ ratio was investigated at a 15 wt % PVDF concentration, resulting in a total of four ESP dopes: ENM10, ENM12, ENM15, and ENM15* (Table 1). After the components fully dissolved, the dope solution was degassed at room temperature for 24 h and used within 1 week for membrane preparation. We did not observe any signs of dope modification during its preparation, storage, or use in electrospinning that could affect nanofiber morphology.

**Table 1:** PVDF Electrospinning Dope Overview

Dope solution	[PVDF] (wt %)	LiCl ratio (mg_LiCl_ g_PVDF_^–1^)	Solvent (v/v)
ENM10	10		6:4 DMF:acetone
ENM12	12		6:4 DMF:acetone
ENM15	15		6:4 DMF:acetone
ENM15*	15	0.43	6:4 DMF:acetone

The viscosity (μ, cP) of the ESP dopes was measured with a Brookfield Programmable DV-II+ Viscometer equipped with a LV Spindle set (Brookfield Engineering Laboratories., Inc., USA) under a rotary motion of 2.5 rpm at 21 °C. A 1% variability in viscosity was assumed, according to the equipment specifications.

The nonwoven PVDF ENMs were fabricated in a Spinbox electrospinner (Bionicia, Spain), equipped with an anodized aluminum rotary drum (20 cm wide and 10 cm diameter, at 400 rpm) covered with aluminum foil to collect the membrane sample. The ESP process and setup conditions for ENM production were a horizontal single static 20 Ga stainless-steel needle, a tip-to-collector distance of 12 cm, a dope flow rate of 1.20 mL h^–1^, and an ESP time of 8 h. At the beginning of each electrospinning run, the applied voltage was incremented until Taylor’s cone became stable. Low voltage led to a dope accumulation at the needle tip until its weight made it fall onto the electrospinner base. However, high voltages led to splaying or branching (dissipation of the Taylor’s cone into multiple fiber streams),^73^ causing excessive drying and clogging of the needle. For each dope solution composition, a tailored voltage in the range of 8.0 to 12.0 kV was selected to ensure a stable jet throughout the entire electrospinning session.

### 2.2 Drying and Heat Treatment

Each ESP run produced a membrane sheet with approximate dimensions of 8 × 28 cm. The ENMs were dried overnight at 85 °C, and then subjected to a heat treatment (HT), in which the ENM was placed between two glass plates and heated in an oven at a specified temperature and time (150 °C for 6h at 70 Pa of pressure corresponding to the weight of the upper plate). The purpose of HT was to establish preliminary cohesion among the nanofibers hot pressing.

### 2.3 Hot Pressing Procedure

To further ensure fiber consolidation and improve membrane integrity, heat-treated membranes were processed in a laboratory HP machine (QIXING Laboratory Mini Hot Press, Wuhan Qien Science & Technology Development CO., LTD; China). The ENMs were hot-pressed at temperatures (T_HP_) of 25, 60, 80, and 120 °C; pressures (P_HP_) of 1.0, 10, and 20 MPa, and hot-pressing times (t_HP_) of 2, 4, 6, and 10 min. To avoid damaging the membranes due to high-temperature decompression, a 4 min isobaric cooling time was implemented after processing, ensuring the sample temperature was below 40 °C.

In two distinct experiment sets, the study elucidated how the HP parameters affected the ENMs hydrophobicity and morphology. The first set determined the temperature and pressure effect on the properties of ENM10 samples over a specified time (6 min). In the second set, the effects of time at different temperatures on the characteristics of ENM15* samples were assessed at a fixed pressure of 10 MPa.

### 2.4 Membrane Characterization Techniques

The thickness of each membrane sample was measured at 20 different points using a micrometer (543-705B Mitutoyo Digital Indicator ID-C, Mitutoyo, Japan) to determine the average thickness and its standard deviation. The dimensionless compression ratio (C_R_) (eq 1) was calculated as the ratio between the average thickness before (*δ*_*o*_, μm) and after HP (δ, μm). The average of partial derivatives error estimated this parameter uncertainty.

1 $$C_{R}=\frac{\delta }{\delta_{o}}$$

Membrane surface density (σ, mg cm^–2^) or the average amount of PVDF per unit of membrane area was calculated using eq 2, where *m*_*M*_ (mg) and *A*_*M*_ (cm^2^) represent the mass and the area of the sample. A laboratory analytical balance (Denver Instrument SI-114, USA) weighed 2.7 × 2.7 cm membrane samples.

2 $$\sigma =\frac{m_{M}}{A_{M}}$$

Membrane hydrophobicity was determined through the sessile-drop water contact angle (WCA) measurement method. A syringe pump (NE-300 KF Technology, USA) dispensed a drop onto the membrane surface. After a 15-s stabilization period, a digital microscope (Celestron Hand-held Digital Microscope Pro, China) captured images of the drops in front of a white light source (Philips HUE Lamp, The Netherlands). The Contact Angle plugin of the ImageJ software processed the images based on the ellipse approximation to determine the WCA. The average and standard deviation were obtained from measurements in five different locations.

The ENMs surface and cross-section morphology images were captured with a Field Emission Scanning Electron Microscope (FESEM) (Hitachi S4800, Hitachi Ltd., Japan), applying a 10 kV accelerating voltage. Prior to analysis, an SC7640 sputter-coater deposited a thin palladium–gold alloy layer on the ENM samples (1 min, 1.8 kV). From the SEM images, the average nanofiber diameter (d_F_, nm) was measured using the ImageJ software from 150 randomly selected fibers. Likewise, the membrane surface porosity (ε_S_) was calculated from two SEM images at 2000× magnification. Results were reported along with their respective standard deviations.^25^

Calorimetric data were obtained using a Setaram Setline+ DSC (Caluire-et-Cuire, France) to investigate the thermal behavior and crystallinity of PVDF electrospun membranes following post-treatment. Samples (∼4 mg) were placed in 30 μL aluminum crucibles and subjected to heating/cooling cycles between 30 and 200 °C, with heating at a constant rate of 10 °C min^–1^ and cooling at 5 °C min^–1^. All experiments were conducted under a nitrogen atmosphere at a flow rate of 50 mL min^–1^ in duplicate. Melting temperatures (*T*_m_) and enthalpies (Δh_m_) were averaged, and peak profile characteristics were used to assess differences in crystalline uniformity and thermal stability induced by the post-treatments. To determine the total crystallinity degree of the samples (χ_c_), the area under the melting peak (Δh_∞_, J g^–1^) was compared with the enthalpy of fusion for a 100% crystalline material (Δh_∞_ for PVDF = 104.6 J g^–1^).^74–76^

The membrane pore size distribution was determined by capillary flow porometry according to the ASTM F316–03 standard.^77,78^ N-dodecane and Porefil served as wetting liquids, with solvent-air surface tensions (γ) of respectively 0.02491 N m^–1^ (at 25 °C) and 0.01637 N m^–1^ (at 20 °C). Briefly, a 25 mm diameter membrane sample was immersed in the solvent and subjected to 70 rpm orbital shaking at room temperature for at least 1 h. Then, the sample was inserted into a sealed filter holder connected to a manometric air pressure (ΔP) controller. A gas flowmeter (Whisper MW Series Meter, Alicat Scientific, USA) measured the wet volumetric flow (F_W,_ L min^–1^) under increasing pressure until the membrane was dry, and the dry volumetric flow (F_D,_ L min^–1^) under decreasing pressure (dry flow, F_D,_ L min^–1^) to obtain the wet and the dry flow curves, respectively. Filter flow (F_F_) (%) was calculated in intervals using eq 3, where “h” and “l” refer to the upper and lower pressure values of each interval. This flow was then related to the pore size (*D*, μm) using Washburn’s eq (eq 4), with contact angles (θ) of 0°. A shape factor (*SF*) of 0.715 was also assumed, based on elliptical pore geometry, which resembled the complex pore architecture of ENMs the most.^51,59^

3 $$F_{F}\left(\%\right)=\left[{\left(\frac{F_{W}}{F_{D}}\right)}_{h}-{\left(\frac{F_{W}}{F_{D}}\right)}_{l}\right]\times 100$$

4 $$\Delta P=\frac{4\cdot \cos\theta \cdot \gamma \cdot SF}{D}$$

An atomic force microscope (AFM) determined the ENMs surface roughness operating in tapping mode (OmegaScope, HARIBA Scientific, Japan) with 125-μm long AFM probes at a constant force of 42 N m^–1^ (PPP-NCHR, Nanosensors, Switzerland). The Gwyddion data analysis software (Department of Nanometrology, Czech Metrology Institute, Czech Republic) processed the membrane topography of 5 × 5 μm scans to determine the root-mean-square roughness (R_q_, nm). The mean R_q_ and its standard deviation were calculated from at least three different membrane surface images.

### 2.5 Hydraulic Stability, Gas–Liquid Operation Performance, and Long-Term Endurance

Membrane integrity and resistance of the fabricated ENMs were first assessed under operating conditions in a flat sheet membrane module, as previously described.^13^ A 6.5 cm diameter ENM was placed in the membrane module and a constant water flow rate of 21 L h^–1^ was applied in the liquid side of the membrane in a closed loop with a peristaltic pump (Percon N-M-II, JP Selecta, Barcelona, Spain). After 24 h of operation without water breakthrough in the gas side of the module, the dissolved CH_4_ recovery experiments started.

The lab-scale setup for dissolved CH_4_ recovery tests comprised a gas–liquid flat membrane contactor, a 2 L feed tank, and a saturation column as main elements, and other auxiliary elements such as water peristaltic pump, mass flow controller and valves (Scheme 1). A full description of the system is available in our previous work.^13^ First, the setup operated in CH_4_ saturation mode, and then in CH_4_ recovery mode, denoted by a red dash line and a black continuous line, respectively. After 1 h of operation under CH_4_ saturation mode, the liquid stream reached a constant CH_4_ concentration of 16 ± 1 mg L^–1^. Then, the dissolved CH_4_ recovery experiment started at a 21 L h^–1^ water flow rate, which was continuously recirculated to the feed tank in a closed loop. A 1.0 L h^–1^ N_2_ stream served as a sweeping gas in crossflow configuration to recover the dissolved CH_4_ through the permeate side. The unsteady-state CH_4_ recovery experiments were conducted for 5 h at 24 °C.

**Scheme 1:**
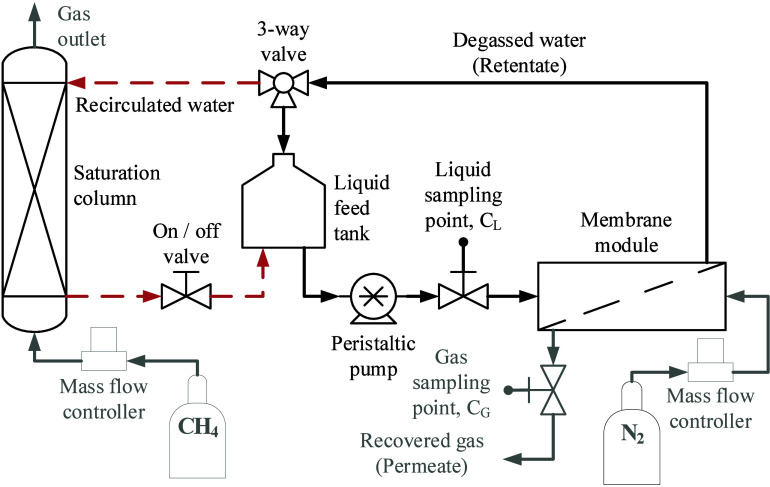
CH_4_ Saturation and Recovery Process Flow Diagram

The dissolved CH_4_ liquid concentration was determined by gas chromatography using the headspace method. In each experiment, 5 mL samples were collected in triplicate every hour from the liquid sampling point into sealed 16 mL vials prefilled with air. Achieving phase equilibrium involved orbital shaking at 25 °C and 200 rpm for 30 min. Then, 0.5 mL of headspace gas was injected into a gas chromatograph (Varian GC CP-3800, USA) equipped with a HAYESEP Q packed column and a thermal conductivity detector to measure the gas phase CH_4_ concentration. The CH_4_ liquid-phase concentration was determined from Henry’s Law, and then both the gas and liquid phase concentrations were used to determine the dissolved CH_4_ concentration in the liquid sample (C_L_, mg L^–1^).^24^

Process assessment involved dissolved CH_4_ removal efficiency (RE_CH4_,%) as a key performance indicator (eq 6). Here, C_L,0_ and C_L,t_ (mg L^–1^) represent dissolved CH_4_ liquid feed tank concentrations at time zero and time (t), respectively.

6 $$RE_{CH4}=\frac{C_{L,o}-C_{L,t}}{C_{L,o}}\cdot 100$$

To assess the long-term operation performance of the ENMs, a long-term stability test was performed with a continuous water flow rate of 21 L h^–1^ for a time on stream (TOS, hours) of 80 h. A control dissolved methane recovery test of 5 h was conducted as described previously, at different TOS of 30, 42, and 80 h. After 80 h of accumulated operation, the membrane was dried at 60 °C and characterized based on membrane thickness, WCA, SEM images and DSC analysis.

## 3 Results and Discussion

### 3.1 Characterization of Membranes Fabricated at Different Dope Compositions after Heat Treatment at Low Pressure

The PVDF electrospun nanofiber membranes (ENMs) fabricated with different ESP dopes (Table 1) were characterized after the heat treatment (HT) at 70 Pa and 150 °C for 6h, to evaluate the effect of the dope solution composition on the ESP voltage, mean nanofiber diameter, WCA, membrane thickness and membrane surface density.

The ESP voltage increased with polymer concentration, with values of 8.1, 8.3, and 9.3 kV for PVDF concentrations of 10, 12, and 15 wt %, respectively, possibly due to the need of a more energetic electric field to maintain Taylor’s cone when processing more viscous ESP dopes.^34,79^ Furthermore, adding LiCl increased the ion concentration in the solution environment and formed charged complexes with the solvent, and the polymer. This interaction increased viscosity, requiring stronger electric fields to overcome electrostatic attractions.^34^ Consequently, the ENM15* dope, with a viscosity of 2180 ± 20 cP, required the highest voltage for processing, at 10.8 kV, whereas the ENM10 dope, with a viscosity of 410 ± 4 cP, required the lowest processing voltage, at 8.1 kV.

Table 2 summarizes the properties of the ENMs, which serve as the initial reference to evaluate the properties change after the HP posttreatment. In the SEM images, the ENMs showed continuous, uniform, and defect-free fibers, regardless of the polymer dope concentration and the presence of LiCl. The free space predominates over the volume occupied by fibers, characteristic of this type of membrane. Also, no sintering regions were observed despite the HT processing (70 Pa and 150 °C for 6 h).

**Table 2:**
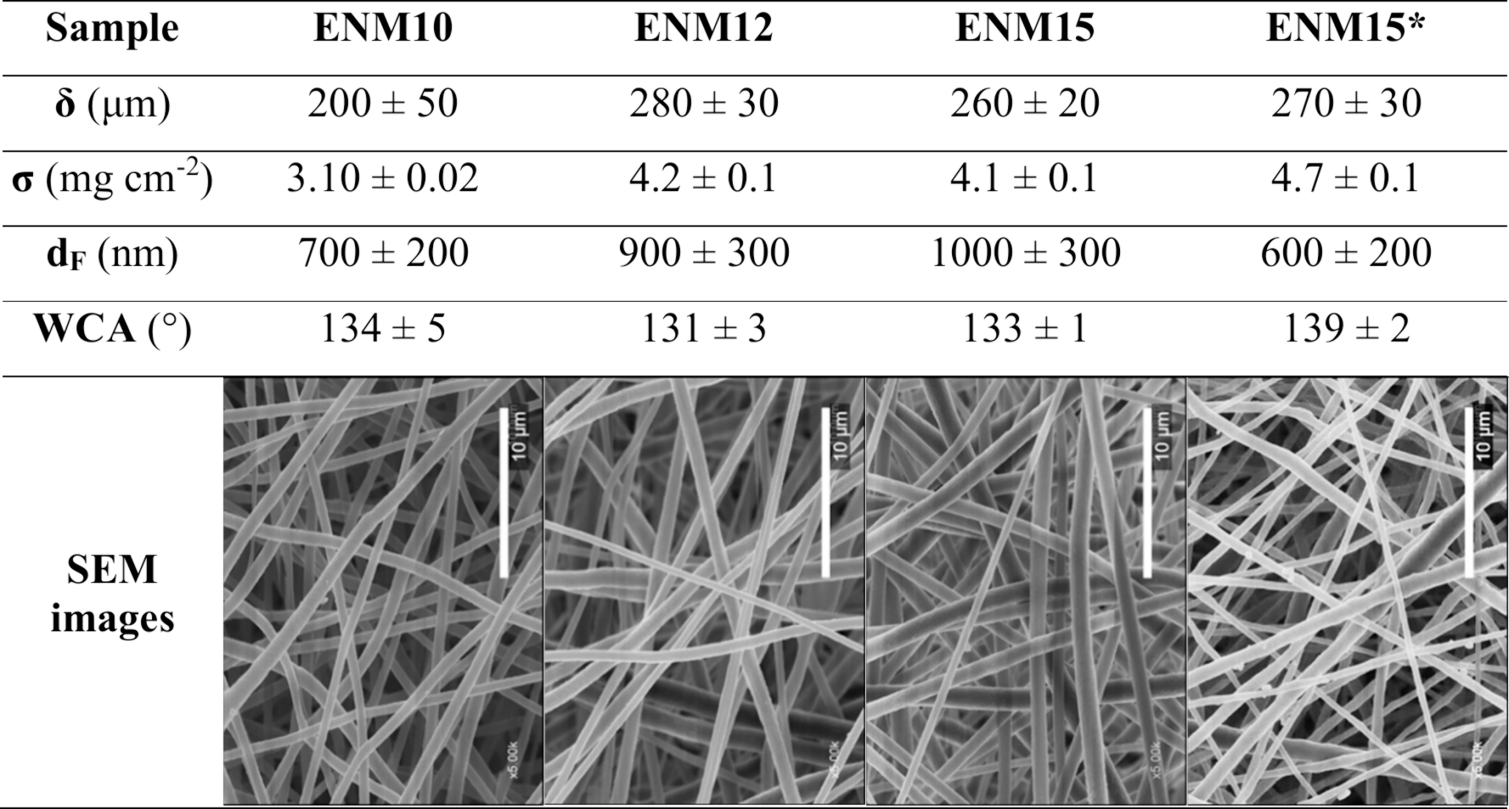
Effect of the Polymer and LiCl Concentration on the Membrane Thickness (δ), Surface Density (σ), Mean Fiber Diameter (d_F_), Water Contact Angle (WCA), and SEM Images of PVDF Electrospun Nanofiber Membranes after Heat Treatment (150 °C, 6 h, 70 Pa)

For LiCl-free ENMs, the average fiber diameter was directly proportional to the PVDF concentration, measuring 700 ± 200 nm and 1000 ± 300 nm for 10 and 15 wt % PVDF, respectively (Table 2). This trend was attributed to increased viscosity, which added resistance to the stretching induced by the electric field.^34^ The addition of LiCl to 15 wt % PVDF dopes resulted in a reduction in average fiber diameter from 1000 ± 300 nm to 600 ± 200 nm. This effect was attributed to the increased electrical conductivity of the solution and the enhanced surface charge density of the polymer jet, which promoted greater jet elongation due to electrostatic repulsion. Subsequently, this effect extended the time the nanofibers remained in the instability region, reducing fiber diameter.^34^ Therefore, the inclusion of LiCl, combined with the increased dope concentration, accelerated the membrane fabrication process, without altering the fiber morphology of the ENMs. This is evidenced by the increased thickness and the surface density of ENM15* compared with ENM10 under the same fabrication time.

High hydrophobicity is desired to prevent membrane wetting during operation. The WCA of samples subjected to HT at 150 °C and 6 h (Table 2) were around 133°, regardless of the PVDF dope concentration. However, introducing LiCl led to a WCA of 139 ± 2°, positively impacting membrane hydrophobicity, which could be attributed to its lower average fiber diameter that contributed to a higher roughness.^80^

Table 3 compiles the results of other researchers in the production of membranes using ESP dopes based on several PVDF with different solvents, including DMF and alternatives such as 1-methyl-2-pyrrolidinone (NMP), dimethyl sulfoxide (DMSO), and dimethylacetamide (DMAc). The PVDF concentration in their ESP dopes ranged from 5 to 28 wt %, with this wide span owing to differences in the molecular weight of the commercial PVDF brands. The previously reported ENMs exhibited fiber diameters ranging from 150 nm^45^ to 1300 nm,^81^ although values typically fell between 400 ^82,83^ and 600 nm,^81,83^ similar to the values reported in this work. Regarding the hydrophobicity nature of the ENMs, WCA values ranged between 115°^45^ and 146°,^82^ though most of the values were between 125°^46^ and 135°,^68^ as the ones reported in this work (Table 2).

**Table 3:** Compilation of Literature Results for PVDF Electrospun Nanofiber Membrane Fabrication: Dope, Fabrication Parameters, Properties, and Applications^a^

Dope formulation and characteristics						Electrospinning conditions for membrane fabrication						Membrane properties					
PVDF brand	[PVDF]	Solvent	Additive	κ (μS cm^–1^)	μ (cP)	TCD (cm)	Q_ESP_ (mL h^–1^)	V (kV)	Collector	Needle size and motion	Postdrying consolidation	δ (μm)	WCA (deg)	d_F_ (nm)	Pore size (nm)	Apl.	Ref
Sigma-Aldrich PVDF-HFP (110 kDa)	15 wt %	68 wt %DMF	None		541	10	1.0	20	Rotary	23 Ga	None	≈100	138	330 ± 80	Avg: 540	MD	^82^
		17 wt %Acet							500 rpm						Max: 1040		
		40 wt % DMF	5 wt % PDMS		1719								146	360 ± 40	Avg: 580		
			40 wt % THF												Max: 1220		
Kynar 761	10 wt %	DMF/Acet				18	0.8	11.5	Rotary	22 Ga	None	10.6	129 ± 4	400 ± 100	N/A	PZTs	^83^
		6:4 v.%							250 rpm							Filtr.	
		DMSO/Acet										19.3	130 ± 2	600 ± 100	N/A		
		6:4 v.%															
		NMP/Acet										15.0	116 ± 2	500 ± 300	N/A		
		6:4 v.%															
Kynar HSV 900	5 wt %	DMF/Acet	0.004 wt % LiCl	46.27	260	12	N/A	27	Rotary	N/A	None	N/A	138	N/A	Avg: 180	MD	^68^
		6:4 wt %							N/A	0.10 mm/s					Max: 360		
											Glass-plate HP: 170 °C, 1 h	N/A	136	N/A	Avg: 210		
															Max: 330		
Solef 6012	10 wt %	DMAc/Acet	None	0.8 ± 0.1	94.6 ± 0.5	15	1.0	–0.125	Flat.	20 Ga	Glass-plate HP: 130 °C, overnight.	N/A	N/A	N/A	N/A	MD	^45^
		6:4 wt %	4.3 mg_LiCl_/g_PVDF_	375 ± 3	107.2 ± 0.4					2D motion		32 ± 6	117 ± 6	N/A	710 ± 60		
			4.3 mg_LiCl_/g_PVDF_	394 ± 6	108.1 ± 0.7							48 ± 1	115 ± 4	150 ± 10	750 ± 40		
			8.3 mg_Ru(phen)3_/g_PVDF_														
Solef 6012	8 wt %	DMSO/Acet	None		79.4	15	1.0	15	Flat.	Size N/A	24h-immersion in water	49 ± 2	123 ± 3	N/A	2290 ± 30	Filtr.	^46^
		6:4 v.%								2D motion	Oven at 40 °C overnight						
		DMF/Acet	0.43 wt % LiCl		87.3							69 ± 1	125 ± 2	N/A	810 ± 40		
		6:4 v.%															
		DMSO/Acet	0.43 wt % LiCl		74.4							73 ± 1	125 ± 2	N/A	900 ± 80		
		6:4 v.%	None		79.4						Glass-plate HP: 130 °C, overnight	35 ± 1	128 ± 1	N/A	2260 ± 80		
			0.43 wt % LiCl		74.4							54 ± 2	127 ± 4	N/A	1270 ± 20		
Kynar K-761	15 wt/v%	DMAc/Acet				15	2.0	15	Flat	0.21 mm	“Post-heat” treatment at 145 °C for 18h	300	145	400 ± 100	400–1060	Filtr.	^84^
		1:1 v.%								Motion N/A							
Sigma-Aldrich (275 kDa)	20 wt %	DMAc			0.95	15	0.6	20–35	Rotary	21 Ga	HP apparatus:	30–40		230 ± 50		PZTs	^81^
	25 wt %				2.1				1500 rpm		60 MPa, 80 °C, 1 h			500 ± 100			
	28 wt %				2.5									800 ± 200			
	22 wt %	DMAc/Acet			1.4									1300 ± 400			
		1:2 v.%															
Kynar 761	10 wt %	DMF/Acet			410 ± 4	12	1.2	8.1	Rotary	20 Ga	Glass-plate HT: 70 Pa, 150 °C, 6h.	200 ± 50	134 ± 5	700 ± 200	700–3500	G-L sep.	This work
	15 wt %	6:4 wt %	0.43 mg_LiCl_/g_PVDF_		2180 ± 20			10.8	400 rpm.	Fixed	Glass-plate HT: 70 Pa, 150 °C, 6 h	270 ± 30	139 ± 2	600 ± 200	900–2800		
	15 wt %		0.43 mg_LiCl_/g_PVDF_								HP: 10 MPa, 60 °C, 6 min	38 ± 6	110 ± 5	700 ± 200	200–800		

a [PVDF]: PVDF concentration. κ: Conductivity (μS cm^–1^). μ: Dynamic viscosity (cP). TCD: Tip-to-collector distance. Q_ESP_: Electrospinning dope flow rate. V: Electric tension. δ: Membrane thickness. WCA: Water contact angle. d_F_: Average fiber diameter. Apl.: Application. Ref.: Reference. Acet: Acetone. PDMS: Polydimethylsiloxane. THF: Tetrahydrofuran. DMF: *N,N*-Dimethylformamide. DMSO: Dimethyl sulfoxide. DMAc: *N*,*N*-Dimethylacetamide. NMP: 1-Methyl-2-pyrrolidone. LiCl: Lithium chloride. N/A: Not available, not reported. 2D: Two-dimensional. MD: Membrane distillation. Ru­(phen)_3_: Tris­(phenantroline)­ruthenium­(II) chloride. HP: Hot pressing. G-L Sep.: Gas–liquid separations. PZTs: Piezoelectrics. Filtr.: Filtration.

The ENMs reported in this study had thickness and polymer surface density values respectively ranging from 200 to 280 μm and from 3.10 to 4.7 mg cm^–2^ (Table 2). Similar results have been reported for PVDF ENMs for particle filtration under lab-scale operational conditions,^84^ though other authors produced membrane thicknesses below 100 μm. The increased membrane thickness in the ENMs of this work was necessary to ensure the integrity of the ENMs for testing in the membrane contactor for the dissolved CH_4_ recovery from water.

### 3.2 Membrane Characterization after Hot Pressing for Membrane Integrity Consolidation

#### 3.2.1 Effect of Hot-Pressing Temperature and Pressure on Membrane Properties

When tested in the flat-sheet membrane contactor under operating conditions with water, the heat-treated ENMs (150 °C, 6 h, 70 Pa) failed. Water was detected on the gas side of the membrane, and the loss of integrity of the membrane nanofibers was also evident as fraying. To overcome this limitation, the ENM consolidation was studied with the application of HP at temperatures (T_HP_) of 25, 60, 80, and 120 °C, and pressures (P_HP_) of 1, 10, and 20 MPa, at a fixed time (t_HP_) of 6 min. Figure 1 summarizes the WCA, compression ratio, and surface membrane porosity results for the ENM10 samples. Surface SEM images are presented in Figure 2, along with the HT-ENM10 sample for comparison purposes.

**Figure 1:**
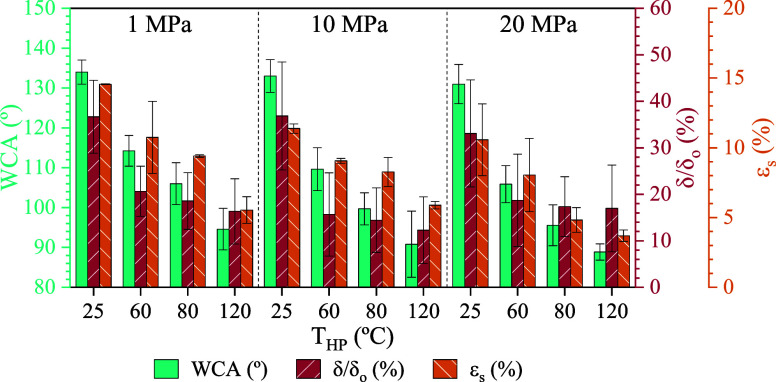
Effect of hot-pressing pressure (P_HP_) and temperature (T_HP_) on water contact angle (WCA), compression ratio (δ/δ_o_), and surface porosity (ε_S_) of 10 wt.% PVDF electrospun nanofiber membrane (ENM10) samples. t_HP_ = 6 min.

**Figure 2:**
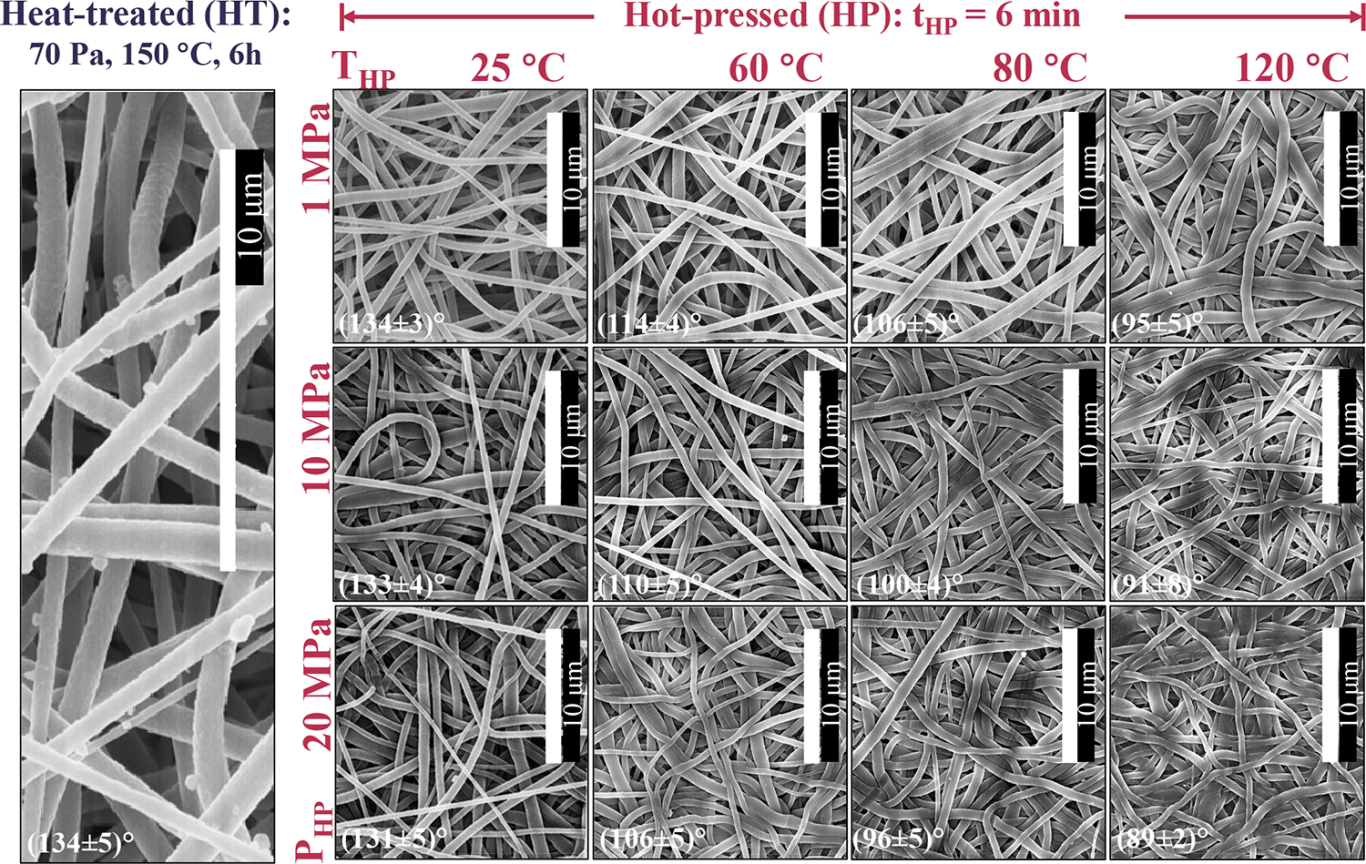
Effect of hot-pressing pressure (P_HP_) and temperature (T_HP_) on surface morphology and water contact angle (WCA) of 10 wt.% PVDF electrospun nanofiber membranes (ENM10). t_HP_ = 6 min.

Regarding the effect of T_HP_, SEM images at the lowest T_HP_ of 25 °C showed randomly oriented fibrous structures with numerous interstices, similar to the initial ENM sample after the HT (150 °C, 6h, 70 Pa) (Figure 2). At 25 °C, surface changes were not detectable even at a P_HP_ as high as 20 MPa, indicating that pressure alone was insufficient to produce remarkable surface changes. As a result, the WCA resulted in values of around 134° at 25 °C for any pressure, like the initial ENM sample. With increasing T_HP,_ WCA decreased, showing negligible variation across different P_HP_. The average WCA values across the examined P_HP_ were 133°, 110°, 100°, and 92° for T_HP_ of 25, 60, 80, and 120 °C, respectively (Figure 1). The reduction in WCA was attributed to the gradual loss of the surface roughness of the ENMs at higher T_HP._ At 120 °C, the WCA reached values near the hydrophilicity threshold (90°), which is undesirable for membrane contactor applications involving gas recovery from aqueous streams. WCA values of HP ENMs obtained in this work at T_HP_ of 60 and 80 °C were comparable to PVDF commercial membranes (Table 4).

**Table 4:** Properties of Several Microporous Commercial Flat-Sheet Membranes^24,25^

Membrane name	Material	Support	WCA (deg)	Pore size (μm)	Thickness (δ, μm)
Durapore (Merck)	PVDF		120 ± 2	0.22^a^	125^a^
PVDF membrane (Dorsan Filtration)	PVDF	PET	103 ± 2	0.20^a^	159 ± 2

a Provided by the supplier.

Qualitatively, SEM images revealed a more compact morphology with increasing T_HP_, characterized by flatter fibers, a higher density of fibers within the same focal plane, fewer voids, and the appearance of sintered regions, especially over 60 °C. These findings were coherent with the surface porosity reduction at increased P_HP_ or T_HP_; samples hot-pressed at 20 MPa and 120 °C exhibited surface porosities of 3.7 ± 0.4%, compared to 23.7 ± 0.3% for the ENMs sample without HP (Figure 1).

The membrane thickness decreased remarkably with the HP posttreatment, resulting in a compression ratio of about 38% at 25 °C for any P_HP_ in the 1–20 MPa range. Membrane thickness was unaffected by P_HP_ variations across the 1 – 20 MPa range at a fixed T_HP_. We observed that the membrane compression ratio was inversely proportional to the T_HP_. The compression ratios were 35%, 18%, 17%, and 15% for T_HP_ of 25, 60, 80, and 120 °C, respectively (Figure 1).

Differential scanning calorimetry (DSC) was conducted to assess possible changes in crystallinity induced by the HT and HP protocols. First heating scans (Figure 3a) showed that the melting temperature (*T*_m_) and melting enthalpy (Δh_m_) remained nearly constant across all samples (166.3 ± 0.6 °C and 42 ± 1 J g^–1^, respectively), indicating no significant chain scission or thermal degradation associated with HT or HP membrane consolidation. However, differences in the melting peak profile were observed. Samples subjected to HT and HP at 1 MPa exhibited signs of a bimodal endotherm, indicative of heterogeneous crystalline domains. Positively, samples hot-pressed at 10 and 20 MPa displayed a sharper, unimodal peak around the main melting peak, implying a more uniform crystalline structure. Indeed, the total degree of crystallinity (χ_c)_ was unaffected by post-treatment, consistently ranging between 40 and 42% across all studied samples, in line with the high thermal stability of PVDF. Cooling behavior (Figure 3b) supported this interpretation, as the crystallization onset temperature (*T*_c_^onset^) and the crystallization enthalpy (Δh_c_) also remained stable (146.7 ± 0.6 °C and −30 ± 1 J g^–1^, respectively) regardless of the post-treatment. The sample subjected to the mildest treatment conditions exhibited a broader and less intense crystallization peak, indicating a more heterogeneous crystalline structure, which sharpened after HP at higher pressure.

**Figure 3:**
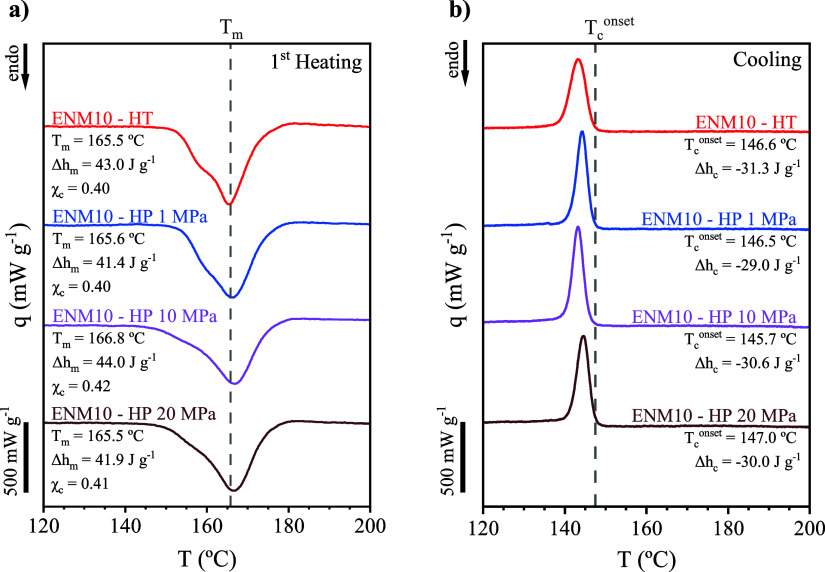
(a) First heating and (b) cooling DSC scans of 10 wt.% PVDF electrospun nanofiber membranes (ENM10) after heat treatment (HT: 150 °C, 70 Pa, 6 h) and hot pressing (HP: 120 °C, 1–10–20 MPa, 6 min).

The effect of T_HP_ on membrane properties was further investigated to evaluate its impact on the pore size distribution. Results are shown in Figure 4 and Table 5 after HP at 10 MPa at different temperatures, and compared with the HT-ENM (150 °C, 6h, 70 Pa). The initial ENM showed a broad pore size distribution in the 1–3 μm range and a negligible pore population with sizes below 1 μm. After the HP at 10 MPa and 25 °C for 6 min, the pore size frequency in the range 0.3 – 1.0 μm raised to around 58% while reducing the frequency of pores in the range 1 – 1.8 μm to around 20%. Increasing the T_HP_ to 60 °C shifted the pore size mode toward 0.2 – 0.8 μm, with a 67% frequency peak at 0.4 μm. For 80 °C HP, the pore size distribution narrowed down to the 0.2 – 0.5 μm range, and the 0.4 μm peak increased its frequency up to 79%. Full analysis of the membrane subjected to a T_HP_ of 120 °C was not possible due to the setup pore detection limit, however, pore sizes below 0.2 μm were detected. The increasing T_HP_ caused the pore size distribution to narrow toward smaller ranges, while particularly favoring the formation of a characteristic pore size of high frequency at each temperature, especially between 60 and 80 °C, where most probable sizes of 0.4 μm were found. The reduction in the ENMs pore size caused by HP was attributed to interfiber compaction and fusion. Other authors have reported that HP enhances the ENMs tensile strength and modulus,^85,86^ making them suitable for applications requiring fine filtration and mechanical stability.^71,85,86^ Therefore, previous studies report pore sizes ranging from 0.2 to 1.0 μm for PVDF ENMs used in filtration and MD applications^45,68,82,84^ (Table 3). Similarly, the pore size distribution of the fabricated ENM with HP post-treatment was comparable to the mean pore size of commercial PVDF membranes commonly used for dissolved gas separation from liquids (Table 4).

**Figure 4:**
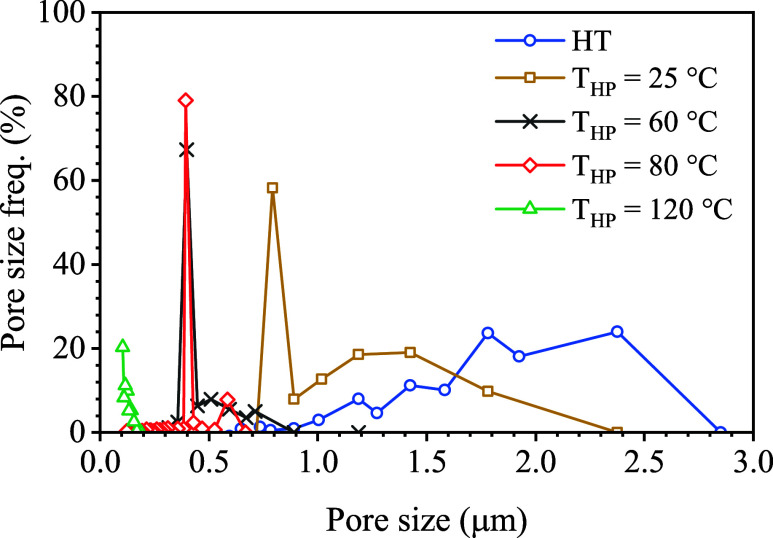
Pore size distribution estimation by capillary porometry for 15 wt.% PVDF/LiCl electrospun nanofiber membrane (ENM15*), heat treated (HT) at 150 °C for 6 h, 70 Pa, and hot-pressed (HP) at P_HP_ = 10 MPa and different temperatures (T_HP_) (t_HP_ = 6 min). Lines were added to the plot to aid visualization.

**Table 5:** Pore Size and Roughness Analysis Results of 15 wt.% PVDF/LiCl Electrospun Nanofiber Membrane (ENM15*), Heat-Treated (HT, 150 °C for 6 h, 70 Pa) and Hot-Pressed at 10 MPa and Different Temperatures (t_HP_ = 6 min; R_q_: Root Mean Square Roughness)

	Pore size (μm)				Roughness (nm)
T_HP_ (°C)	Min.	Max.	Mode	Mode freq (%)	R_q_
HT	0.71	2.85	2.37	24	N/A
25 °C	0.71	2.38	0.79	58	400 ± 100
60 °C	0.19	0.89	0.40	67	360 ± 90
80 °C	0.12	0.67	0.40	79	280 ± 30
120 °C	<d.l.	0.19			250 ± 90

The enhanced membrane compactness of the HP ENMs at 10 MPa with the increasing T_HP_ was confirmed with the cross-section SEM images presented in Figure 5, along with the ENM without HP. Above 60 °C, the fibers began to sinter, leading to fewer and smaller pores. In other words, at this higher-pressure range (1–20 MPa), fiber fusion begins between 60 and 80 °C, a significantly lower T_HP_ than that required in the HT, where around 170 °C was required for the porous structure to arrange into a more compact form.^68^ Yet, in all cases, and although increasingly compressed at higher temperatures, the unique electrospun nanofiber architecture is preserved after 10 MPa HP. These results were coherent with the capillary porometry analysis (Figure 4 and Table 5), where the 0.4 μm pore size frequency peak was found to increase from 67% at a T_HP_ of 60 °C, to 79% at a T_HP_ of 80 °C, which is indicative of a transition toward a reduced interstitial space in the nanofibrous structure, which explains why pore sizes above 0.2 μm were absent at a T_HP_ of 120 °C.

**Figure 5:**
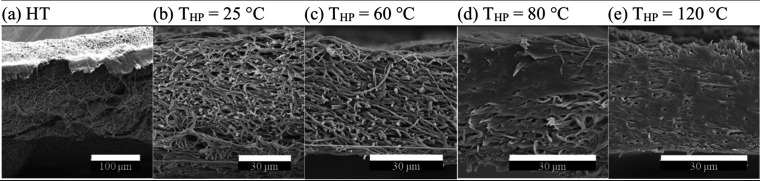
Effect of heat treatment (HT) and hot-pressing temperature (T_HP_) on the SEM cross-section morphology of 15 wt.% PVDF/LiCl electrospun nanofiber membranes (ENM15*). HT at 70 Pa, 150 °C, 6 h. P_HP_ = 10 MPa, t_HP_ = 6 min.

ENMs roughness results (Table 5, Figure 6) showed a reduction in R_q_ with increasing T_HP_ from 400 ± 100 nm at 25 °C and 250 ± 90 nm at 120 °C. These results agree with the SEM image analysis and the decrease in ENMs WCA at higher T_HP_ by Wenzel’s effect, which states that roughness amplifies the natural tendency of a surface to repel or attract water.^87^ AFM analysis of non-HP ENM samples was hindered by the frequent presence of prominent peaks and valleys in the topography and by low cohesion between fibers, suggesting that its roughness was much higher than that of the HP ENMs at 10 MPa. The reduction in roughness at higher T_HP_ was coherent to the greater degree of consolidation, the reduced distance between fibers, and, consequently, the decreasing pore size of the sample.

**Figure 6:**
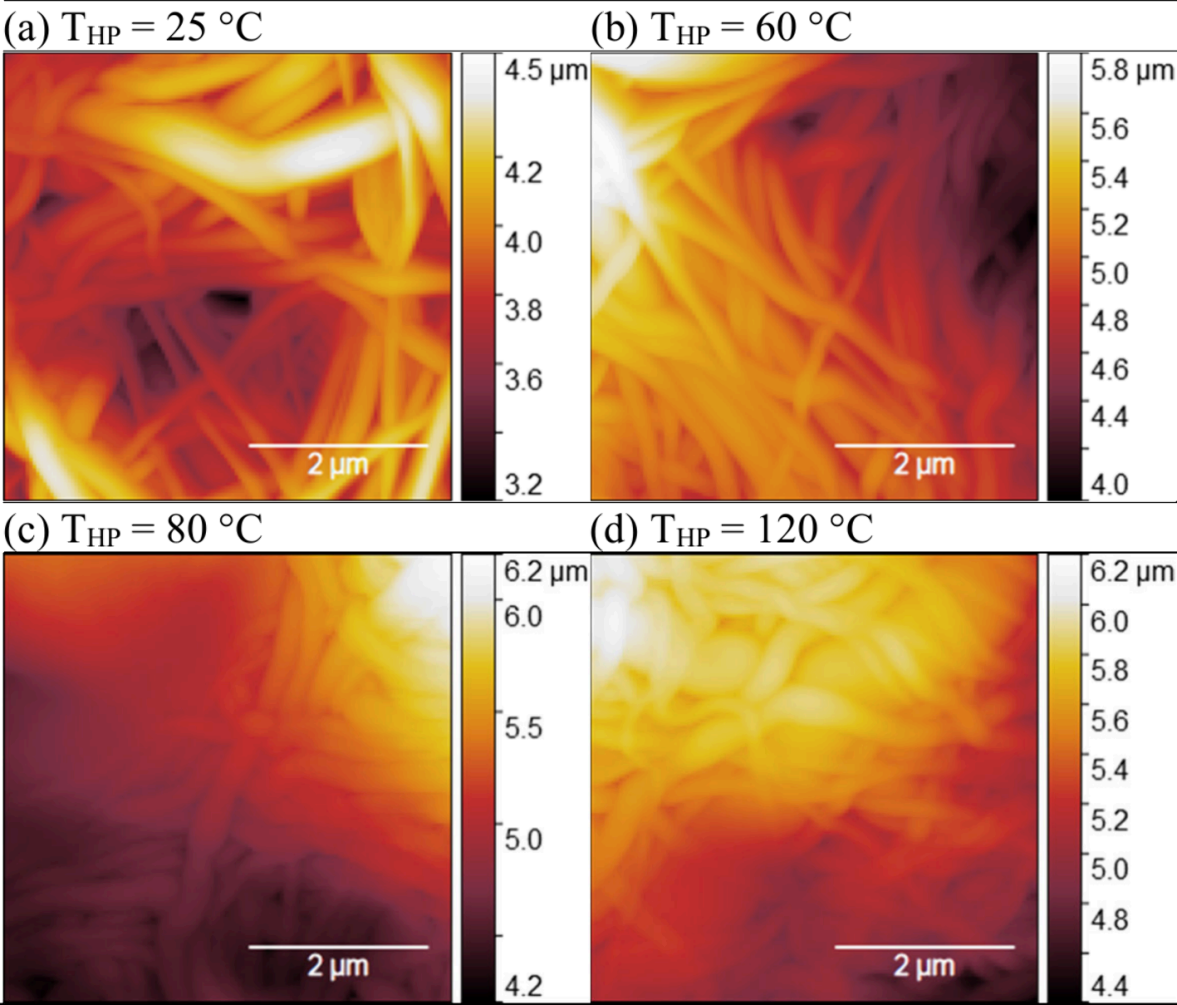
Effect of hot-pressing temperature (T_HP_) on the surface topography of 15 wt % PVDF/LiCl electrospun nanofiber membranes (ENM15*) at a P_HP_ = 10 MPa and t_HP_ = 6 min. Atomic force microscopy (AFM) images.

#### 3.2.2 Effect of Hot-Pressing Time on the Membrane Properties

The effect of the hot-pressing time (t_HP_) on membrane thickness, compression rate, surface density, and WCA was evaluated in the 2 – 10 min t_HP_ range at different T_HP_ (60, 80, and 120 °C) at 10 MPa of P_HP_, and results were shown in Figure 7 compared along with the ENM15* subjected only to HT at 150 °C for 6h (t_HP_ = 0 min). The 2–10 min range includes typical t_HP_ found in the literature for PVDF ENMs processing.^71,88,89^ This study found that these membrane properties remained nearly constant with the increase in t_HP_ from 2 to 10 min.

**Figure 7:**
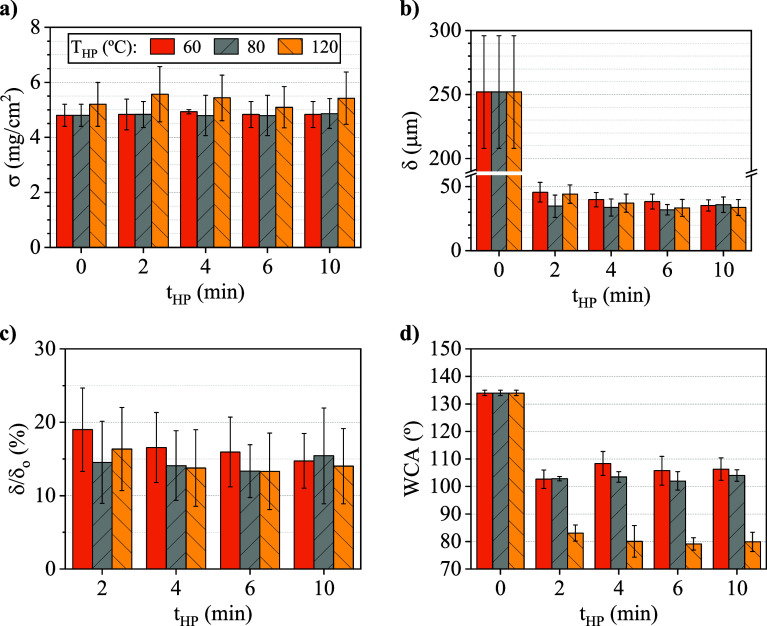
Effect of hot-pressing time (t_HP_) at different temperatures (T_HP_) on (a) membrane surface density, (b) average thickness, (c) compression ratio (δ/δ_o_), and (d) WCA of 15 wt.% PVDF/LiCl electrospun nanofiber membrane (ENM15*) samples. P_HP_ = 10 MPa (t_HP_ = 0 min corresponds to the heat-treated ENM without hot pressing).

The initial polymer surface density and thicknesses were of 4.7 mg cm^–2^ and 270 ± 30 μm, respectively. While the polymer surface density remained constant after HP treatment at any t_HP_ and T_HP_ (Figure 7a), membrane thickness reduced to about 40 μm with an average compression ratio of about 16% for all the HP conditions respect to the ENM without HP (t_HP_ = 0 min) (Figure 7b and 7c). Compression caused the fiber diameter to slightly increase; in ENM15* samples without HP, the fiber diameter was 600 ± 200 nm, but after 10 MPa and 60 °C HP, they expanded to 700 ± 200 nm. Kaur et al. (2011) also reported a decrease in thickness with increasing P_HP_, and an increment in fiber diameter after HP PAN fibers at 87 °C for 999 s under P_HP_ exceeding 0.14 MPa, attributed to fiber sintering.^41,67^ These indicators confirm that the HP treatment successfully compacted the ENMs without causing significant mass loss, as the sample mass remained consistent before and after treatment, nor did it lead to any surface expansion.

Regarding hydrophobicity (Figure 7d), WCA decreased by 20% at 60 and 80 °C under 10 MPa compared to the ENMs without HP (t_HP_ = 0 min). At 120 °C, the reduction reached 40%, causing the ENMs to fall below the 90° hydrophobicity threshold, thereby limiting their application in gas–liquid membrane contactors, as previously stated. The results also suggest that while HP influenced hydrophobicity, WCA remained constant for t_HP_ ranging from 2 to 10 min.

Yang et al. studied the effect of 5, 25, 40, and 60 min of t_HP_ at 400 kPa, 200 °C on polyacrylonitrile (PAN) ENMs as one of the steps to produce carbon paper for electrode applications. They found that the ENMs retained their morphology and density on all time settings. However, t_HP_ influenced the specific surface area, crystallinity, and concentration of defects in the resulting carbon paper, which in turn altered their properties for electrode applications.^90^ To the best of our knowledge, no reports are currently available that study the effect of t_HP_ on PVDF ENMs for filtration or gas–liquid applications.

### 3.3 Performance Test: Evaluation of the Dissolved CH_4_ Removal Efficiency from Water

The HP PVDF ENMs were tested in operation in a flat-sheet membrane contactor for the removal of dissolved CH_4_ from water. The HT PVDF ENMs (150 °C, 6 h, 70 Pa) failed under operational conditions with water in the flat-sheet membrane contactor, since water was detected in the gas side during operation, probably due to the presence of larger pores with sizes of 1–3 μm pores on these membranes (Figure 4a). In contrast, the HP ENM (10 MPa, 60 °C, 6 min), having most pores around a size of 0.4 μm, withstood the operational conditions required for gas–liquid operation in the membrane contactor for up to 24 h, so the dissolved CH_4_ recovery test was conducted and performance results were presented in Figure 8, along with results obtained with a commercial PVDF membrane.

**Figure 8:**
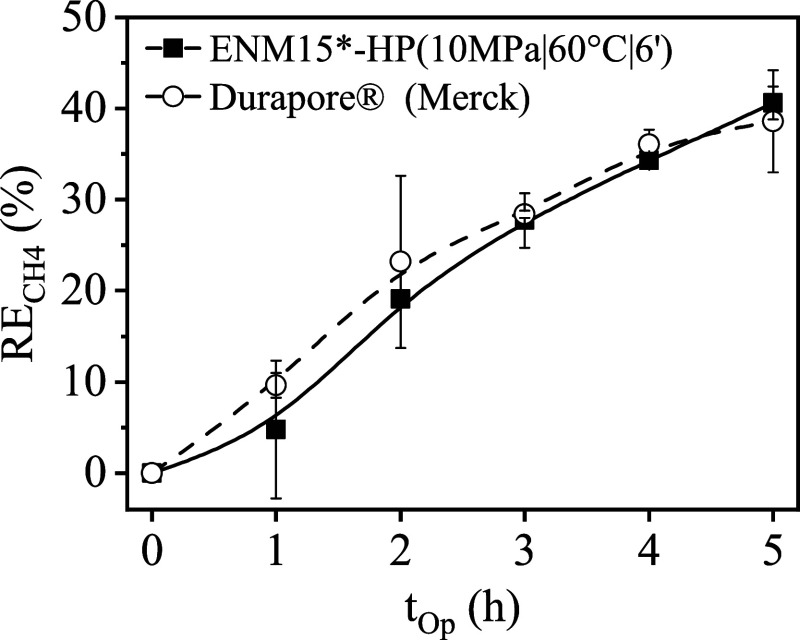
CH_4_ removal efficiency (RE_CH4_) vs operating time (t_Op_). Benchmarking for commercial and hot-pressed electrospun membrane.

A dissolved CH_4_ recovery of 40% at 5 h of operation in the nonstationary process was observed with the HP ENM15*, matching the performance of the commercial PVDF membrane. Similar recovery efficiencies of 40–45% have been reported under similar operating conditions after 5 h of operation with commercial PDMS, PP, and PVDF membranes.^24^ These results demonstrated that the ENM consolidation under HP conditions enabled the fabrication of PVDF ENMs with appropriate mechanical resistance and stability in operation, complying with the current efficiency standards of commercial membranes.

### 3.4 Long-Term Endurance and Postservice Characterization

After the initial methane recovery test described in the previous section with the HP ENM (TOS = 30 h), the membrane was kept in the module under the 21 L h^–1^ water flux and two dissolved methane recovery tests of 5 h of operation (t_Op_) were repeated at different TOS (42 and 80 h) to evaluate the RE_CH4_. Results presented in Figure 9 showed that the RE_CH4_ at 3 h of operation resulted in 27.2, 25.0 and 24.1% at TOS of 30, 42, and 80 h, respectively. A similar slightly declining trend was observed at 5 h of operation, with RE_CH4_ of 40.6, 39.6%, and 37.0%, respectively. Although these variations could be attributed to experimental error, the consistent decline suggests a gradual increase in mass transfer resistance over time, likely due to changes in the membrane’s structure or surface properties during extended operation.

**Figure 9:**
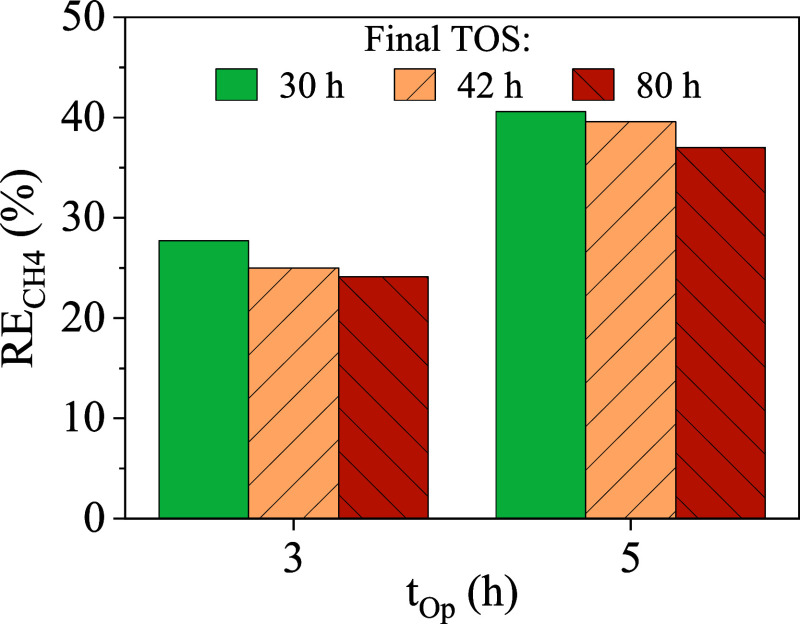
CH_4_ removal efficiency (RE_CH4_) vs operation time (t_Op_) at different time on stream (TOS).

Comparison of membrane photographs before and after the long-term testing indicated signs of both surface wear and partial wetting (Figure 10). Transparent regions were observed after use (TOS = 80 h), possibly indicating areas of water penetration. These regions disappeared after drying (Figure 10c), supporting the occurrence of water entry.

**Figure 10:**
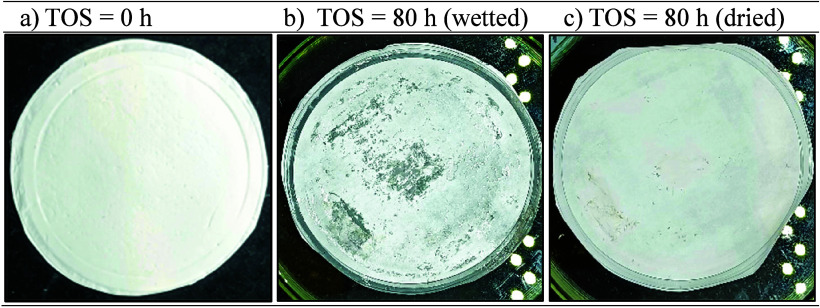
Photographs of the 15 wt.% PVDF/LiCl electrospun nanofiber membrane (ENM15*) sample hot-pressed at 10 MPa and 60 °C during 6 min, (a) before the long-term test (TOS = 0 h), (b) after the test (TOS = 80 h), and (c) after its use and drying at 60 °C (TOS = 80 h, dried).

Surface wear was further evidenced in SEM images (Figure 11). Before the operation test, initially well-bound nanofibers were observed (Figure 11a), which became loose and partly detached from the membrane bulk after a TOS of 80 h (Figure 11b), likely due to continuous shear stress from the liquid flow across the membrane surface.

**Figure 11:**
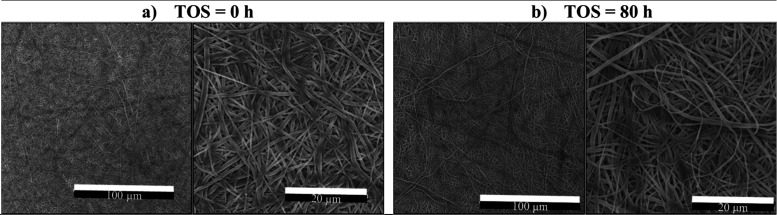
SEM images at different magnifications of the 15 wt.% PVDF/LiCl electrospun nanofiber membrane (ENM15*) sample hot-pressed (HP at 10 MPa, 60 °C, 6 min), (a) before the long-term test (TOS = 0 h) and (b) after the test and drying at 60 °C (TOS = 80 h).

The membrane properties also became more heterogeneous over TOS. The average membrane thickness increased from 40 ± 6 μm to 70 ± 20 μm after a TOS of 80 h, with a notable rise in thickness variability. This change could result not only from fiber detachment but also from membrane decompaction. As the fibrous packing became disrupted, pore enlargement could occur, allowing water to partially enter the membrane structure.

The average WCA varied from 108 ± 4° (WCA in the range 116° ± 127°) before use to 100 ± 10° (WCA in the range 87° ± 115°) after 80 h of operation. This variation was consistent with the increased surface heterogeneity observed in postoperation.

Calorimetric analyses were also performed to the membrane after a prolonged TOS of 80 h. The results showed a negligible impact on its crystalline structure. The DSC scans showed heating and cooling profiles nearly identical to its analogous counterpart (Figure 3), so the thermal and crystallinity parameters were very similar (*T*_m_ = 166.3 °C, Δh_m_ = 42.8 J g^–1^, χ_c_ = 41%, *T*_c_^onset^ = 147.3 °C, Δh_c_ = −34.0 J g^–1^).

## 4 Conclusions

The combined use of electrospinning (ESP), and postprocessing heat treatment (HT) and hot pressing (HP) produced hydrophobic PVDF membranes for gas–liquid separations, which were tested for dissolved CH_4_ recovery from water.

Concerning electrospinning, the increase of PVDF concentration from 10 to 15 wt % improved the ESP performance but enlarged fiber diameter; adding LiCl helped to control fiber size and enhanced hydrophobicity, resulting in membranes with WCA ∼ 140° and ∼ 270 μm thickness after HT with glass plates at 70 Pa and 150 °C for 6 h. However, these membranes failed in the flat-sheet membrane contactor operation due to broad pore size distribution and low fiber consolidation, causing water breakthrough and integrity loss.

At more severe pressing conditions, HP effectively consolidated the nanofibers, narrowed and reduced pore sizes, and preserved the PVDF total crystallinity. HP of the ENM at 60 – 120 °C, 1–20 MPa, and 2–10 min showed that high temperature lowered WCA by reducing surface roughness, while pressure and pressing time had minimal additional effects.

The resulting hot-pressed ENM matched commercial PVDF membranes in hydraulic stability and dissolved methane recovery efficiency, maintaining stable performance for at least 80 h.

## Data Availability

Data is available on Zenodo, 10.5281/zenodo.15622613.

## ABBREVIATIONS

**Acronym/Abbreviation**: **Meaning**
2D: Two-dimensional
Acet.: Acetone
AFM: Atomic force microscopy
Apl.: Application
DMAc: N,N-dimethylacetamide
DMF: Dimethylformamide
DMSO: Dimethyl sulfoxide.
DSC: Differential scanning calorimetry
ENM(s): Electrospun nanofiber membrane(s)
ESP: Electrospinning
FESEM: Field emission scanning electron microscope
Filtr.: Filtration
G-L: Gas–liquid
G-L Sep.: Gas–liquid separations
HP: Hot pressing/hot-pressed
HT: Heat treatment/heat-treated
MC: Membrane contactors
MD: Membrane distillation
N/A: Not available, not reported
NMP: 1-Methyl-2-pyrrolidone
PAN: Polyacrylonitrile
PDMS: Polydimethylsiloxane
PVDF: Polyvinylidene fluoride
PZTs: Piezoelectrics
ref.: Reference
SF: Shape factor
TCD: Tip-to-collector distance
THF: Tetrahydrofuran
tos: Time on stream
WCA: Water contact angle
**SYMBOLS**
**Symbol**: **Meaning**
[PVDF]: PVDF concentration
A_M_: Membrane sample area
CH_4_: Methane
C_L,o_: Dissolved CH_4_ liquid concentration at time zero
C_L,t_: Dissolved CH_4_ liquid concentration at time “t”
C_R_: Compression ratio
CO_2_: Carbon dioxide
D: Pore size
d_F_: Average nanofiber diameter (nm)
F_D_: Dry flow (L min^–1^)
F_F_: Filter flow (%)
F_W_: Wet flow (L min^–1^)
LiCl: Lithium chloride
LiNO_3_: Lithium nitrate
m_M_: Membrane sample mass
P_HP_: Hot pressing pressure
Q_ESP_: Electrospinning dope flow rate (mL h^–1^)
RE_CH4_: Dissolved-methane removal efficiency
R_q_: Root mean square roughness (nm)
Ru­(phen)_3_: Tris­(phenanthroline) ruthenium­(II) chloride
t: Time
*T*_c_^onset^: Crystallization onset temperature
T_HP_: Hot pressing temperature
*T*_m_: Melting temperature
t_HP_: Hot pressing time
t_Op_: Operating time
V: Electrospinning operating voltage/electric tension
γ: Surface tension (N m^–1^)
δ: Membrane thickness after hot pressing
δ/δ_o_: Compression ratio
δ_o_: Membrane thickness before hot pressing
Δh_c_: Crystallization enthalpy
Δh_m_: Melting enthalpy
Δh_∞_: Enthalpy of fusion for a 100% crystalline material
ΔP: Manometric pressure
ε_s_: Membrane surface porosity
θ: Contact angle (deg)
κ: Conductivity (μS cm^–1^)
μ: Dynamic viscosity (cP)
σ: Membrane surface density (mg cm^–2^)
χ_c_: Total crystallinity degree (%)
